# Supplementary material for: Prevalence and Demographic Correlates of Substance Use among Adults with Mental Illness in Eastern Cape, South Africa: A Cross-Sectional Study
Source: Int J Environ Res Public Health. 2021 May 19;18(10):5428. doi: 10.3390/ijerph18105428 (PMC8161045; doi:10.3390/ijerph18105428)
Supplement: Supplementary file 1 [file ijerph-18-05428-s001.zip › ijerph-1153107-supplementary.pdf]

International Journal of
Environmental Research and Public Health

MDPI

Table S1. Common Diagnoses.

.	Frequencies	Percentages
Schizophrenia	159	40.8
Major depressive symptoms	72	18.5
Cannabis-related	75	19.2
Bipolar	69	17.7
Alcohol-related disorder	39	10.0
Psychotic disorder	26	6.7
Neurocognitive disorder	18	4.6
Stimulant use disorder	14	3.6
Sedative hypnotic anxiolytic	16	4.1
Moderate intellectual disability	15	3.8
Antisocial borderline personality disorder	10	2.6
Mild intellectual disability	10	2.6
Generalized anxiety disorder	8	2.1
Post-traumatic stress disorder	5	1.3
Autism spectrum disorder	4	1.0
Cocaine-related disorder	3	0.8
Other substances	3	0.8
Other specified schizophrenia spectrum	3	0.8
Unspecified schizophrenia spectrum and psychotic disorder	3	0.8
Tobacco related	2	0.5
Severe intellectual disability	2	0.5
Opiod related disorder	1	0.3
Sleep–wake disorders	1	0.3

Table S2. Relationship between common mental health disorders and risky alcohol and psychoactive drug use.

Diagnosis.	Past Year Risky Alcohol Use		Past Year Psychoactive Drug Use	
	N (%)	*p*-Values	N (%)	*p*-Values
Schizophrenia Spectrum and other Psychotic Disorders	34 (18.2)	0.891	31 (16.6)	0.668
Depressive Disorders	8 (11.1)	0.075	9 (12.5)	0.222
Bipolar and Related Disorders	11 (15.9)	0.552	6 (8.7)	0.035
